# Acid Hydrolysis of Quinoa Starch to Stabilize High Internal Phase Emulsion Gels

**DOI:** 10.3390/gels10090559

**Published:** 2024-08-28

**Authors:** Songnan Li, Chaohui Sun, Ye Sun, Enpeng Li, Ping Li, Jun Wang

**Affiliations:** 1 Sericultural & Agri-Food Research Institute Guangdong Academy of Agricultural Sciences, Key Laboratory of Functional Foods, Ministry of Agriculture and Rural Affairs, Guangdong Key Laboratory of Agricultural Products Processing, Guangzhou 510610, China; lsnyz2020@yzu.edu.cn; 2 Joint International Research Laboratory of Agriculture and Agri-Product Safety of the Ministry of Education of China, Institutes of Agricultural Science and Technology Development, Yangzhou University, Yangzhou 225009, China; 3 Laboratory of Crop Genomics and Molecular Breeding, Key Laboratory of Plant Functional Genomics of the Ministry of Education, Jiangsu Key Laboratory of Crop Genetics and Physiology, Agricultural College, Yangzhou University, Yangzhou 225009, China; mx120220767@stu.yzu.edu.cn (C.S.); 221702420@stu.yzu.edu.cn (Y.S.); lep@yzu.edu.cn (E.L.); 4 Jiangsu Co-Innovation Center for Modern Production Technology of Grain Crops, Yangzhou University, Yangzhou 225009, China; 5 School of Tourism and Cuisine, Yangzhou University, Yangzhou 225127, China

**Keywords:** high internal phase emulsions, high internal phase emulsion gels, starch nanocrystals, acid hydrolysis, quinoa starch, hydrolysis time

## Abstract

Starch nanocrystals (SNCs) to stabilize high internal phase emulsions (HIPEs) always suffer low production efficiency from acid hydrolysis. Due to its small granule size, Quinoa starch (QS) was selected to produce SNCs as a function of acid hydrolysis time (0–4 days), and their structural changes and potential application as HIPEs’ stabilizers were further explored. With increasing the acid hydrolysis time from 1 day to 4 days, the yield of QS nanocrystals decreased from 30.4% to 10.8%, with the corresponding degree of hydrolysis increasing from 51.2% to 87.8%. The occurrence of QS nanocrystals was evidenced from the Tyndall effect and scanning electron microscopy with particle size distribution. The relative crystallinity of QS subjected to different hydrolysis times (0–4 days) increased from 22.27% to 26.18%. When the acid hydrolysis time of QS was 3 and 4 days, their HIPEs showed self-standing after inversion, known as high internal phase emulsion gels (HIPE gels), closely related to their densely packed interfacial architecture around oil droplets, seen on an optical microscope, and relatively high apparent viscosity. This study could provide a theoretical guidance for the efficient production and novel emulsification of SNCs from QS to HIPE gels.

## 1. Introduction

High internal phase emulsions (HIPEs), known as high-concentration emulsions or gel-like emulsions, have internal phase volume fractions exceeding the close-packing limit (~74%), which leads to semi-solid characteristics and high loading capacities for hydrophobic bioactive compounds [1]. They have been explored as substitutes for mayonnaise [2] and inks for 3D food printers [3]. Notedly, high internal phase Pickering emulsions (HIPPEs) stabilized by solid particles (including nano and microparticles) instead of conventional surfactants showed many more advantages including requiring lower amounts of stabilizer, higher stability against coalescence, higher storage stability, and less environmental pollution as compared to HIPEs [4], thus attracting increased research attention. Natural solid particles used in HIPPEs absorb irreversibly at the interface and form rigid protective layers around droplets and produce characteristically stable emulsions [5]. Food-grade HIPPEs produced using particles derived from polysaccharides have been used for specific applications in food, medical, material, and cosmetic products due to their unique properties [6].

As biodegradable and inexpensive natural biopolymers, starch granules are becoming a promising particle stabilizer for HIPEs after modification. Among starch modifications, acid hydrolysis of native starch to obtain starch nanocrystals (SNCs) is an efficient method used in the industry for chemical modification of starch particles [7]. Platelet-like SNCs with a size of 40–100 nm prepared by sulfuric acid hydrolysis of waxy maize starch could form stable O/W emulsions of 50% (*v*/*v*) paraffin liquid [8]. They have further been used to stabilize stable and gel-like HIPEs at a concentration of 1.0% (*w*/*w*) and internal phase of 75–85% (*v*/*v*) soy oil [9]. However, the low yield and time-consuming preparation of SNCs has limited their applications in Pickering stabilizers for emulsions and fillers for reinforcing natural rubber or biodegradable films [10]. Dufresne, Cavaille and Helbert [11] reported that SNCs could be obtained from hydrolyzing potato starch (5%, *w*/*w*) in a 2.2 M HCl suspension at 35 °C for 15 days. SNCs could also be prepared from hydrolyzing waxy maize starch (10%, *w*/*w*) with 3.16 M H_2_SO_4_ at 40 °C for 6 days [8]. The structure and properties of SNCs were affected by the starch source, acid type, concentration, and hydrolysis parameters (temperature and time) [7]. Among numerous starch sources, Quinoa starch (QS) showed a novel potential for SNCs’ production with a higher yield and shorter time due to its small granule size (0.5–3 μm) and low amylose content (7–26%), which could facilitate the acid hydrolysis reaction. Velásquez-Castillo, Leite, Ditchfield, do Amaral Sobral and Moraes [12] investigated different acid hydrolysis temperatures (30, 35 and 40 °C) on the kinetics and physicochemical properties of nanocrystal production and found that QS nanocrystals produced at 35 and 40 °C could be used as reinforcements in nanocomposite materials or as colloidal stabilizing agents.

Although some attempts have been made to obtain QS nanocrystals by acid hydrolysis, detailed investigations on the effect of hydrolysis time on the production efficiency combined with structural changes in QS and their potential in the application of HIPEs have not been conducted, and even fewer have been presented on high internal phase emulsion gels (HIPE gels). Hence, the present work aims to study the production efficiency (yield and degree of hydrolysis) and structural characteristics (particle morphology with size distribution, FT-IR and XRD pattern) of QS nanocrystals from acid hydrolysis as a function of hydrolysis time (0–4 days referred to as QS, QS-1, QS-2, QS-3 and QS-4, respectively). Subsequently, their potential as HIPEs’ stabilizers was investigated from aspects of the emulsions’ appearance, morphology and apparent viscosity, and their possible mechanism for HIPE gels stabilized by QS nanocrystals was further elucidated. This study could provide a theoretical guidance for the efficient production and novel emulsification of QS nanocrystals for HIPE gel applications.

## 2. Results and Discussion

### 2.1. Yield and Degree of Hydrolysis (DH)

**Figure 1:**
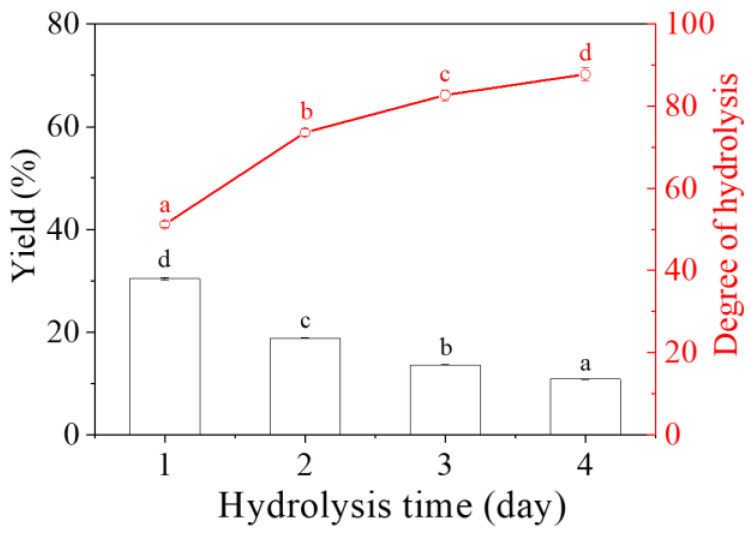
Yield of QS nanocrystals and DH of QS as a function of hydrolysis time (1–4 days). Different superscript letters (a–d) were used to represent statistical significance among different samples (*p* < 0.05).

Figure 1 shows the yield of QS nanocrystals and DH of QS as a function of hydrolysis time (1–4 days). As the hydrolysis time increased from 1 day to 4 days, the yield of QS nanocrystals decreased significantly from 30.4% to 10.8% (*p* < 0.05), which was most certainly a result of the significantly increasing DH from 51.2% to 87.8% (Table S1). Similarly, Velásquez-Castillo, Leite, Ditchfield, do Amaral Sobral and Moraes [12] also found that the yield of QS nanocrystals was 6.8% with a DH of 91.0% at 40 °C on day 5, despite slight differences in acid hydrolysis parameters, mainly including reaction temperature and time. This result was different from that of nanocrystals from other starch sources (normal maize, 14.1%; potato, 8.8%; and tapioca, 15.1%) [13], which should be closely related to the molecular structure of starch. Fast (amylose chains with DP > ~300 and amylopectin long intra-cluster branches) versus slow (amylose chains with DP < ~300) distinct kinetics phases existed for maize, sago and high amylose maize starch during the hydrolysis process, while only a single first-order kinetics phase was involved for that of waxy maize starch [14].

### 2.2. Tyndall Effect

**Figure 2:**
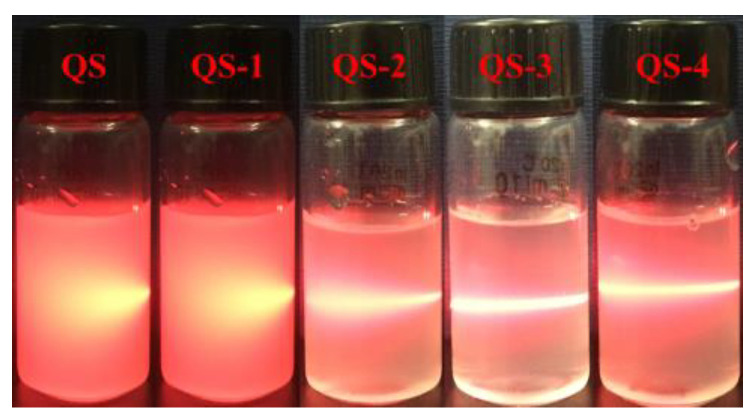
Tyndall effect of QS as a function of hydrolysis time (0–4 days).

Figure 2 exhibits the Tyndall effect of QS as a function of hydrolysis time (0–4 days). For QS and QS-1, the micro-sized starch particles substantially prevented the laser beam from going through the suspension, resulting in a short light path with a conical beam. With an increase in acid hydrolysis time from 2 days, apparent Tyndall light scattering with a brighter and longer light optical path was observed (Figure 2-Q2), implying the presence of nano-sized starch particles. This phenomenon was also observed for waxy maize SNCs with ultrasonic-assisted production [15] and tapioca starch suspensions induced from wet-media milling [16], which is closely related to their nanoparticle size distribution and cluster size of aggregated nanoparticles [17].

### 2.3. Particle Morphology

**Figure 3:**
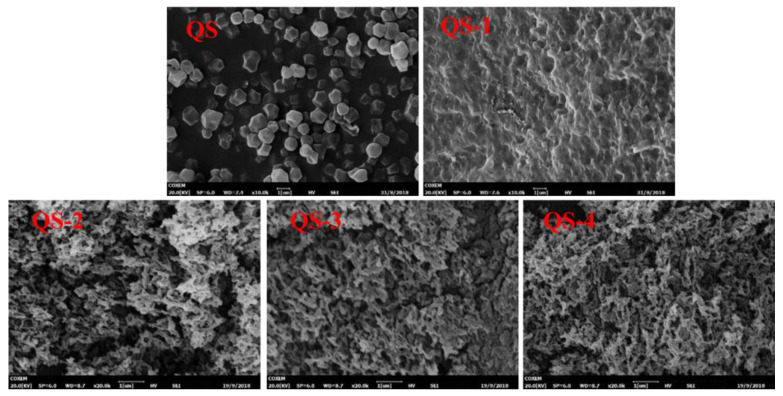
SEM images of QS as a function of hydrolysis time (0–4 days).

Figure 3 presents the SEM images of QS as a function of hydrolysis time (0–4 days). Native QS granules showed polygonal and irregular shapes, consistent with the previous study [18]. After 1 day of acid hydrolysis, the particle size of QS-1 became smaller with a serious aggregated surface as compared to that of QS. Notedly, more nano-sized particles with enormous pores were observed for QS-2, QS-3 and QS-4, resulting from the difference in DH. As reported previously, a low degree of hydrolysis did not significantly alter the granular morphology of starch with a roughened outer surface, extensive hydrolysis resulted in damage to the internal part of starch granules, and more extensive hydrolysis led to the destruction of starch granules into platelet nanocrystals [7]. The particle morphology of SNCs varied with starch sources (different in shape, size, surface features and more fine structures) and degree of acid hydrolysis [19].

### 2.4. Particle Size Distribution

**Figure 4:**
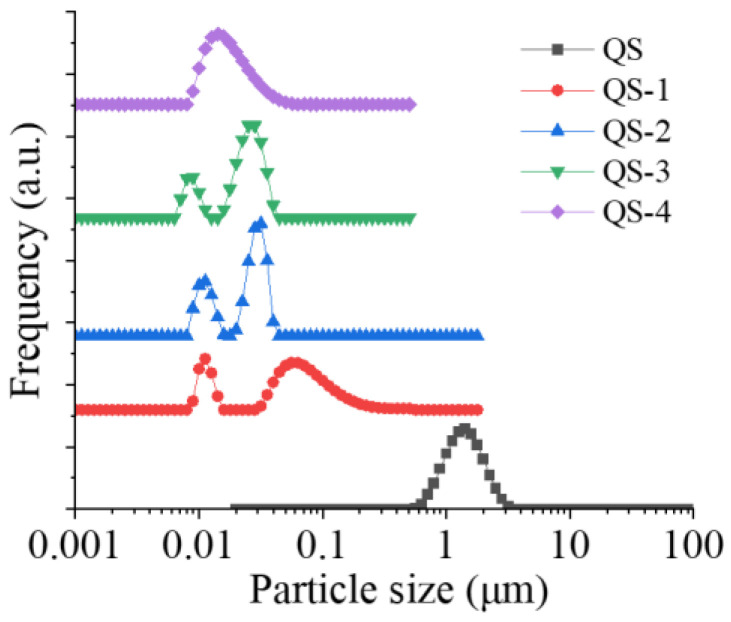
Particle size distribution of QS as a function of hydrolysis time (0–4 days).

Figure 4 presents the particle size distribution of QS as a function of hydrolysis time (0–4 days). There was only one unimodal peak around 1.5 μm for the particle size distribution of QS, similar to the previous study [20]. With the hydrolysis time increasing from 1 day to 3 days, both the main peak around 100 nm and a new peak around 20 nm shifted progressively in the direction of lower particle size, indicating the occurrence of acid hydrolysis [12]. As the hydrolysis time further increased to day 4, only one peak around 20 nm was observed, which may be due to the mostly completed acid hydrolysis, as evidenced by the data of DH. Similarly, a bimodal size distribution around ~100 nm and ~400–900 nm was also reported for QS nanocrystals produced at 35 and 40 °C [12]. The smaller size of QS nanocrystals in comparison to other SNCs from different sources and acid hydrolysis parameters could impart desirable and beneficial properties in nanocomposites.

### 2.5. Fourier Transform Infrared (FT-IR) Pattern

**Figure 5:**
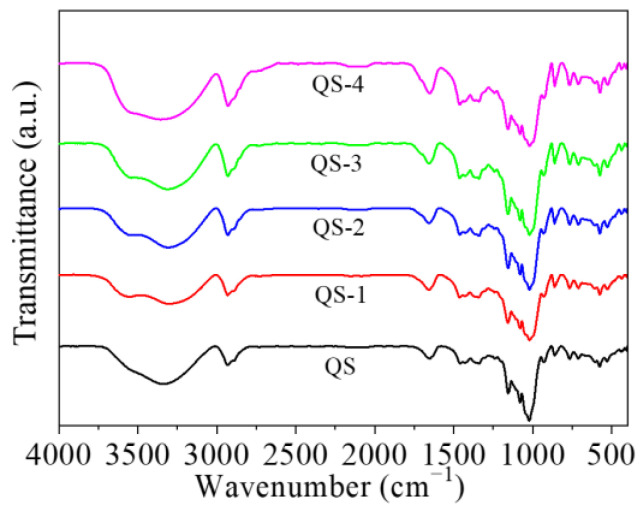
FT-IR pattern of QS as a function of hydrolysis time (0–4 days).

Figure 5 presents the FT-IR pattern of QS as a function of hydrolysis time (0–4 days). Regardless of hydrolysis time, QS nanocrystals showed similar QS FT-IR characteristics, implying the main functional groups of QS remained during the hydrolysis process. Similar results were also reported for nanocrystals acid-hydrolyzed from waxy maize starch [21] and acorn starch [22]. Notably, an increase in intensity in the bands from 3600–3650 cm^−1^ to 3500–3600 cm^−1^ with an increasing hydrolysis time (0–4 days) was observed, which could be ascribed to the hydrolysis-induced changes in the hydrogen bonds of starch molecules [23]. During acid hydrolysis, starch macromolecules broke into small fragments with the breakage of α-1,4 and α-1,6 glycosidic bonds, resulting in enhanced -OH groups [24]. The variation in the region ~900 cm^−1^ could be associated with the disappearance of an amorphous fraction and the presence of more ordered structures [12].

### 2.6. X-ray Diffraction (XRD) Pattern

**Figure 6:**
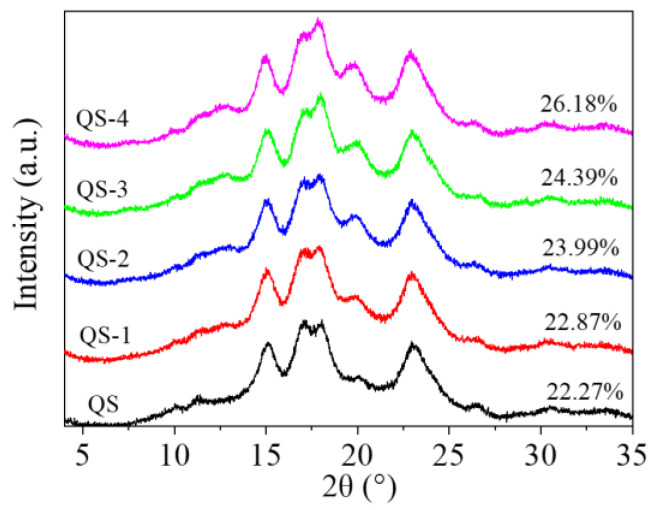
XRD pattern of QS as a function of hydrolysis time (0–4 days).

Figure 6 exhibits the XRD pattern of QS as a function of hydrolysis time (0–4 days). Native QS and QS nanocrystals had an A-type crystalline pattern at ~17°, 18° and 23°. The relative crystallinity of QS subjected to hydrolysis times (0–4 days) increased from 22.27% to 26.18% with an unchanged A-type crystalline pattern. Generally, acid hydrolysis could increase the relative crystallinity of starch granules but it did not change the crystalline polymorphs of A- and B-type starch, although it did change the transition of XRD patterns from A to C or A to B for barley and tapioca starches after acid hydrolysis [7]. During acid hydrolysis of starch, the amorphous region was preferentially damaged due to the water absorption [7], leaving a dense packing of starch chains, responsible for their increased relative crystallinity. Additionally, QS-4 showed a lower relative crystallinity (26.18%) as compared to that of maize starch (~50%) after 10 days of acid hydrolysis [25], which could be due to the difference in the chain length distribution of amylopectin [24]. Regardless of acid hydrolysis parameters, it was too short to rearrange the double helix structure for a high ratio of short-to-long chains and a high percentage of A-chains in the crystalline regions of starch granules [26].

### 2.7. HIPE Appearance

**Figure 7:**
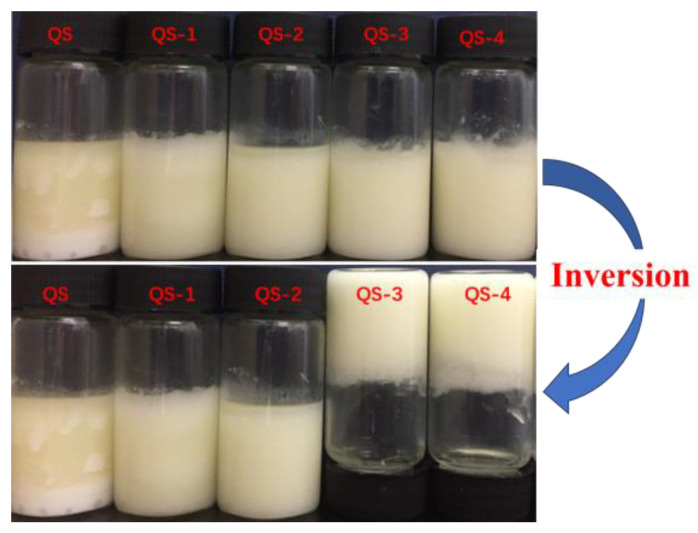
Appearance of HIPEs stabilized by QS as a function of hydrolysis time (0–4 days).

Figure 7 shows the appearance of HIPEs stabilized by QS as a function of hydrolysis time (0–4 days). Native QS was not a good emulsifier for HIPEs due to the severe oiling off at the top phase of fresh emulsions. With hydrolysis time increasing, oiling off was improved with the presence of a white emulsion layer for QS-1 and QS-2. Notedly, the emulsions stabilized by QS-3 and QS-4 were self-standing since they were immobile after inversion, which is a typical feature of emulsion gels, referred to as high internal phase emulsion gels (HIPE gels) [20]. Similar results could be found in the previous reports for SNCs from waxy maize starch [8,9]. Yang, Zheng, Zheng, Liu, Wang and Tang [9] also found that stable and gel-like HIPEs stabilized by SNCs from waxy maize starch could be fabricated at appropriate conditions, such as with high oil phase values of 0.75–0.85, the absence of NaCl, and pH values of 5.0–10.0, with a given particle concentration of 1.0%. Gel-like emulsions or emulsion gels have been widely used in the food, pharmaceutical and cosmetic industries as carriers of bioactive substances and fat substitutes due to their unique three-dimensional droplet structures and elastic–viscous properties [1,27].

### 2.8. HIPE Morphology

**Figure 8:**
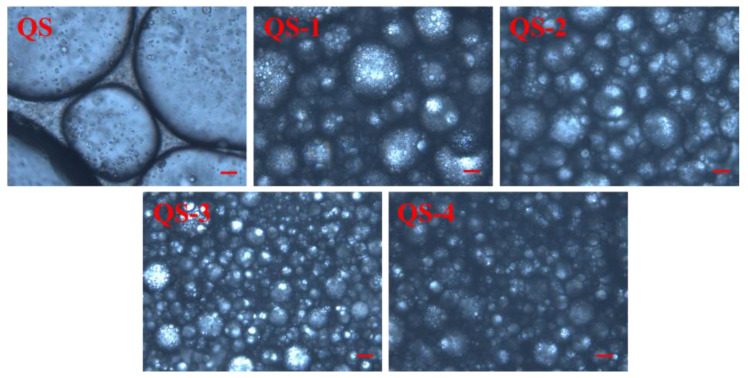
Morphology of HIPEs stabilized by QS as a function of hydrolysis time (0–4 days). (Bar is 10 μm.)

Figure 8 presents the morphology of HIPEs stabilized by QS as a function of hydrolysis time (0–4 days). A poor emulsification with big and irregular oil droplets could be observed for QS; it could be improved obviously by acid hydrolysis, especially for QS-3 and QS-4, resulted from aggregated regular droplets with micron-scale and densely packed interfacial architectures surrounding the droplets. This result should be responsible for the formation of HIPE gels stabilized by QS nanocrystals in Figure 7. Regarding the efficient emulsification concentration for stabilizing HIPEs, QS-4 (1.0%) showed an obvious advantage as compared to SNCs from acorn starch (2.5%) [22] and sago starch (3.5%) [28]. Their varied emulsion morphology could be jointly influenced by particle size, wettability and emulsification parameters [9,29].

### 2.9. HIPE Apparent Viscosity

**Figure 9:**
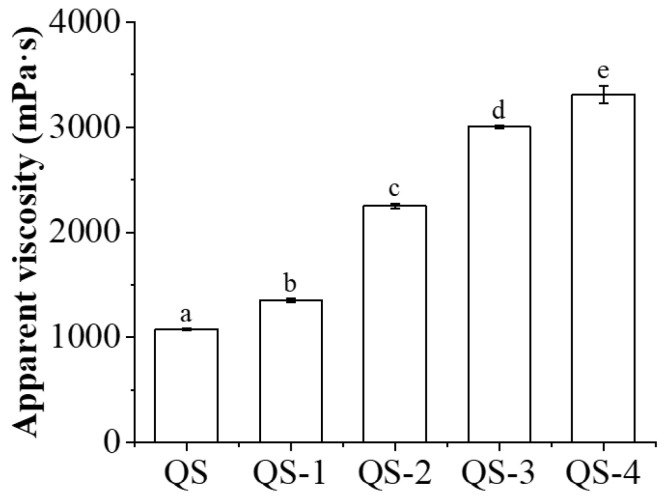
Apparent viscosity of HIPEs stabilized by QS as a function of hydrolysis time (0–4 days). Different superscript letters (a–e) were used to represent statistical significance among different samples (*p* < 0.05).

Figure 9 shows the apparent viscosity of HIPEs stabilized by QS as a function of hydrolysis time (0–4 days). The apparent viscosity of HIPEs stabilized by native QS was 1080 mPa·s, which is lower than that of octenyl succinic anhydride (OSA)-modified waxy maize starch (3918 mPa·s) [30], which should be related to their emulsification performance and parameters (such as concentration and oil fraction) [31]. As the hydrolysis time increased from 1 day to 4 days, their apparent viscosity values increased from 1353.3 mPa·s to 3313.3 mPa·s (Table S2), implying their enhanced droplet network, evidenced by Figure 8. Similar results were observed for starch nanocrystals prepared from ultrasonic-assisted acetic acid [32]. The apparent viscosity could also be affected by the size of the starch molecules, wherein it demonstrated a significantly positive correlation with average whole molecular size, average chain length and degree of substitution for OSA-modified starch [30].

### 2.10. Possible Mechanism for HIPE Gels Stabilized by QS Nanocrystals

**Figure 10:**
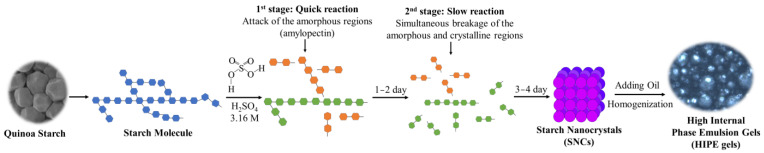
Possible mechanism for HIPE gels stabilized by QS nanocrystals with varying hydrolysis times.

The use of SNCs as stabilizers for HIPEs is highly feasible due to their unique characteristics, including a nano-sized scale and hydrophobic nature, and their active surface resulting from different acid hydrolyses [29]. As reported previously [19,33], the first stage for acid hydrolysis of starch granules is a quick reaction, wherein the amorphous regions mainly consisting of amylopectin are attacked; the following second stage is simultaneous breakage of the amorphous and crystalline regions including amylose and amylopectin. In this regard, the small size of starch granules could be a novel candidate for nanocrystal production with a higher yield and a shorter time. Therefore, QS subjected to different acid hydrolysis times (0–4 days) and their effects on the formation of HIPEs were further explored. As expected, with the increasing hydrolysis time of QS, obvious decreasing trends were observed for yield (Figure 1), particle size (Figure 3 and Figure 4) and relative crystallinity (Figure 6), with increasing DH (Figure 1) and active surface groups (Figure 5). All of the above changes should be responsible for the performance of HIPEs, including typical features of emulsion gels (Figure 7), known as high internal phase emulsion gels (HIPE gels), the densely packed interfacial architecture surrounding oil droplets (Figure 8), and the enhanced apparent viscosity (Figure 9) for QS-3 and 4. Based on the abovementioned results, a possible mechanism for HIPE gels stabilized by QS nanocrystals, with varying hydrolysis times, was proposed in Figure 10.

## 3. Conclusions

This study investigated the effect of hydrolysis time (0–4 days) on the production efficiency and structural changes of QS nanocrystals from acid hydrolysis, and their potential application as HIPE stabilizers was further explored. After 4 days of hydrolysis time, QS-4 showed the highest DH of 87.8%, the lowest yield of 10.8%, the smallest particle size of around 20 nm and the highest relative crystallinity of 26.18%. In addition, QS-3 and QS-4 stabilizing HIPEs were self-standing since they were immobile after inversion, known as high internal phase emulsion gels (HIPE gels), resulting from their densely packed interfacial architecture and aggregated oil droplets. The optimal emulsification parameters, with stability evaluations (such as zeta potential and droplet size changes), for QS nanocrystals and their practical application as HIPE gels in food and cosmetic products is worth further research.

## 4. Materials and Methods

### 4.1. Materials

Quinoa seed was kindly provided by Yuechen Agricultural Technology Co., Ltd. (Xinzhou, China). QS with a purity of 98.6% was obtained using a wet-milling method [18]. H_2_SO_4_ (purity of 98%) was purchased from Sigma Aldrich. Corn oil was obtained from a local supermarket (Yangzhou, China). All other chemicals used in this study were of reagent grade.

### 4.2. QS Nanocrystal Preparation

QS nanocrystals were produced by acid hydrolysis according to a previous report [9] with minor modifications. QS suspension (14.7%, *w*/*v*) was prepared with 3.16 M H_2_SO_4_ solution and incubated at 40 °C with magnetic stirring at 200 rpm for 0–4 days. When the hydrolysis reaction was completed, the above suspension was centrifuged at 10,000 rpm for 10 min, and the supernatant was analyzed to determine the total sugar content using the phenol-sulfuric method. The degree of hydrolysis (DH) was determined by the following equation.

$$DH\;(\%)=\frac{Total\;sugar\;content}{Initial\;dry\;QS\;mass}\times 100$$

The above residue after centrifugation was repeatedly washed with distilled water until it was neutral and then freeze-dried to obtain QS nanocrystals. The yield was calculated by the following equation.

$$Yield\;(\%)=\frac{Freeze-dried\;material\;mass}{Initial\;dry\;QS\;mass}\times 100$$

### 4.3. Tyndall Effect Observation

A sample suspension (2%, *w*/*v*) was prepared for observing the Tyndall effect by a light beam incident from the right side and then photographed [15,34].

### 4.4. SEM Observation

The sample was adhered to the aluminum stub using double-sided carbon tape and then sprayed with gold under vacuum. The microscopic appearance was observed by an SEM (COXEM, Daejeon, Republic of Korea) at an acceleration voltage of 20 kV [20].

### 4.5. Particle Size Determination

The sample was dispersed in deionized water (0.1%, *w*/*v*) with ultrasonic treatment for 3 min and then measured for particle size distribution using a nanoparticle analyzer (HORIBA, SZ-100Z, Tokyo, Japan) according to a previously described method [35].

### 4.6. FT-IR Analysis

The sample with KBr (1:150, *w*/*w*) was pressed into 1 mm pellets for FT-IR (Vector 33, Bruker Optics, Ettlingen, Germany) analysis over the wavenumber range of 400–4000 cm^−1^ [34,35].

### 4.7. XRD Analysis

All the samples were moisture balanced in a desiccator containing saturated BaCl_2_ solution for 3 days before analysis [12]. An X-ray diffractometer (D8 Advance, Bruker, Berlin, Germany) from 4 to 35° 2θ at 0.5 °/min using γ = 0.154 nm Cu K radiation at 40 kV and 30 mA was used, and then the XRD pattern was analyzed by Jade software 6.5 version for the relative crystallinity [18].

### 4.8. HIPE Preparation

The sample suspension (0.2 g; 14.8 mL) was mixed with corn oil (5.2 mL) and homogenized at 20,000 rpm for 1 min using a high-speed shear homogenizer (Heidolph, Schwabach, Germany) to obtain the HIPEs with a concentration of 1.0% and oil fraction of 74% [36]. Aliquots (10 mL) of the above HIPEs were accurately transferred into glass tubes and sealed for further examination.

### 4.9. Appearance and Microscopic Observation

Each fresh HIPE appearance was photographed using a digital camera (EOS 200D II, Canon, Tokyo, Japan). Optical microscopic observation of HIPE was performed using an Olympus BH-2 light microscope (Olympus Co., Tokyo, Japan) [37].

### 4.10. Apparent Viscosity Measurement

The apparent viscosity of freshly prepared HIPEs was measured at 600 rpm of shear speed using a digital rotational viscometer (NDJ-5S, Shanghai Changji Geological Instrument Co., Ltd., Shanghai, China) as described before [30].

### 4.11. Statistical Analysis

All measurements were conducted in triplicate, and the results are presented as mean ± standard deviation. One-way analysis of variance and Duncan’s multiple-range test (*p* < 0.05) were used for significant differences among groups.

## Data Availability

The datasets generated for this study are available on request to the corresponding author.

## Supplementary Materials

The following supporting information can be downloaded at: https://www.mdpi.com/article/10.3390/gels10090559/s1, Table S1: Yield of QS nanocrystals and DH of QS as a function of hydrolysis time (1–4 days) with statistical analysis; Table S2: Apparent viscosity of HIPEs stabilized by QS as a function of hydrolysis time (0–4 days) with statistical analysis.
