# Supplementary material for: Acid Hydrolysis of Quinoa Starch to Stabilize High Internal Phase Emulsion Gels
Source: Gels. 2024 Aug 28;10(9):559. doi: 10.3390/gels10090559 (PMC11430963; doi:10.3390/gels10090559)
Supplement: Supplementary file 1 [file gels-10-00559-s001.zip › gels-3153781-supplementary.pdf]

Supplementary File

Acid Hydrolysis of Quinoa Starch to Stabilize High Internal Phase Emulsion Gels

Table S1 Yield of QS nanocrystals and DH of QS as a function of hydrolysis time (1–4 days) with statistical analysis

Hydrolysis time (day)	Sample	Yield (%)	DH (%)
1	QS-1	30.4 ± 0.2^{d}	51.2 ± 0.7^{a}
2	QS-2	18.8 ± 0.1^{c}	73.6 ± 0.9^{b}
3	QS-3	13.6 ± 0.1^{b}	82.7 ± 1.3^{c}
4	QS-4	10.8 ± 0.1^{a}	87.8 ± 1.6^{d}

Different superscript letters (a–d) were used to represent statistical significance among different samples ($p < 0.05$).

Table S2 Apparent viscosity of HIPEs stabilized by QS as a function of hydrolysis time (0–4 days) with statistical analysis

Sample	Apparent viscosity (mPa·s)
QS	1080.0 ± 10.0^{a}
QS-1	1353.3 ± 15.3^{b}
QS-2	2250.0 ± 26.4^{c}
QS-3	3003.3 ± 15.3^{d}
QS-4	3313.3 ± 83.3^{e}

Different superscript letters (a–e) were used to represent statistical significance among different samples ($p < 0.05$).
